# Relationship between Low Vegetable Consumption, Increased High-Sensitive C-Reactive Protein Level, and Cardiometabolic Risk in Korean Adults with Tae-Eumin: A Cross-Sectional Study

**DOI:** 10.1155/2021/3631445

**Published:** 2021-05-11

**Authors:** Jieun Kim, Kyoungsik Jeong, Siwoo Lee, Younghwa Baek

**Affiliations:** Future Medicine Division, Korea Institute of Oriental Medicine, Daejeon 34054, Republic of Korea

## Abstract

An anti-inflammatory diet has many beneficial effects on cardiometabolic diseases. Constitution type of traditional Korean medicine can predict cardiometabolic risk factors. We examined the relationship between vegetable consumption and the high-sensitive C-reactive protein (hs-CRP) level on cardiometabolic risk factors in Korean adults by constitution types. Data from 1,983 eligible participants (mean age, 44.3 years) were included in the present cross-sectional study. The inflammatory status of the participants was categorized into low- (<3.0 mg/L) or high-risk (≥3.0 mg/L) groups based on their constitution types. Cardiometabolic risk factors (abdominal obesity, elevated triglycerides, reduced high-density lipoprotein-cholesterol, elevated blood pressure, elevated fasting plasma glucose, and ≥2 concurrent cardiovascular diseases (CVDs) risk factors) and dietary assessment of the participants were assessed. A total of 11.1% of Tae-eumin (TE) and 4.9% of non-TE groups had a higher hs-CRP level (TE: 6.6 ± 0.2, non-TE: 8.4 ± 0.3) than a low hs-CRP level TE and non-TE (TE: 0.9 ± 0.1, non-TE: 0.6 ± 0.1). Vegetable consumption of <91.5 g/day was highly associated with a high-risk hs-CRP level (adjusted odds ratio (ORs): second tertile (T2): 2.290, (95% confidence interval (CI): 1.285–4.082); first tertile (T1): 2.474 (95% CI: 1.368–4.475), *P*=0.003) compared with that of the highest (T3) in TE. Low (T1 and T2) vegetable consumption was associated with a 54–63% increased prevalence of more than two concurrent CVDs risk factors compared with that of the highest in the TE group (*P*=0.012). Higher vegetable consumption greatly decreased the prevalence of CVDs risk factors by 63–86% in the low-risk and high-risk hs-CRP TE groups. Our results highlight the cardioprotective effects of higher consumption of vegetables in Korean adults with TE. Evidence-based clinical risk factor management and multifaceted approaches at the community and population levels targeting prevention in high-burden groups are recommended to reduce the premature mortality attributed to CVD.

## 1. Introduction

Obesity-induced complications such as hyperlipidemia, hyperglycemia, and hypertension accelerate a lifelong risk of cardiometabolic multimorbidity [1]. The increasing prevalence of cardiometabolic-related deaths makes it the leading cause of death worldwide in individuals under 65 years of age [2].

High-sensitive C-reactive protein (hs-CRP) determines the inflammatory status, and the existence of inflammation is reported to be related to several features of cardiovascular disease (CVD) [3]. High levels of hs-CRP (>3 mg/L) cause higher relative cardiovascular risk, which is an underlying pathophysiological process in conditions with a chronic inflammatory status such as obesity, type 2 diabetes (T2DM), and CVD [4]. Independent associations have been reported between hs-CRP concentration and hypertriglyceridemia and the reduced high-density lipoprotein-cholesterol (HDL-c) level [5], insulin resistance [6], and cardiovascular events [7].

In traditional Korean medicine, the constitution type of individuals can be identified by their physical and psychological traits, including body structure, function, and metabolism [8]. Constitution type has displayed accuracy in recent studies in predicting metabolic syndrome (MetS) [9] and CVD [10]. Particularly, the Tae-eumin (TE) type showed a positive association with the components of MetS, including obesity-related factors [11–14] and T2DM [15]. Recent cohort studies have reported lower levels of adiponectin and ghrelin hormone, which act as a regulator of food intake [16], and proatherogenesis [17] in TE. Similarly, the obesity-linked response of food and diet in TE has been reviewed [18].

Lifestyle risk factors including smoking, drinking, inactivity, and unhealthy diet considerably increased the prevalence of onset of MetS and CVDs [19]. Among them, dietary preventive measures act as a modifiable risk factor in the inflammatory pathways [20, 21]. A prudent diet high in fruits, vegetables, nuts, and whole grains, which has beneficial effects on the anti-inflammatory capacity by lowering the CRP and CVDs risks, has been recommended in numerous evidence-based studies [22–24].

No study to date has assessed the relationship between dietary factors, hs-CRP concentration, and cardiometabolic risk factors according to constitution types. Therefore, we aimed to elucidate the association of beneficial or harmful dietary factors, serum hs-CRP levels, and cardiometabolic risk factors in Korean adults according to their constitution types.

## 2. Materials and Methods

### 2.1. Study Design and Population

The Korean Medicine Daejeon Citizen Cohort (KDCC) study [25] is the first prospective ongoing cohort study to assess the associations with lifestyle factors and chronic diseases based on the type of traditional Korean medicine. In the present study, we only analyzed the cross-sectional survey data after obtaining the permission of the Korean Medicine Data Center (KDC) administration.

**Figure 1:**
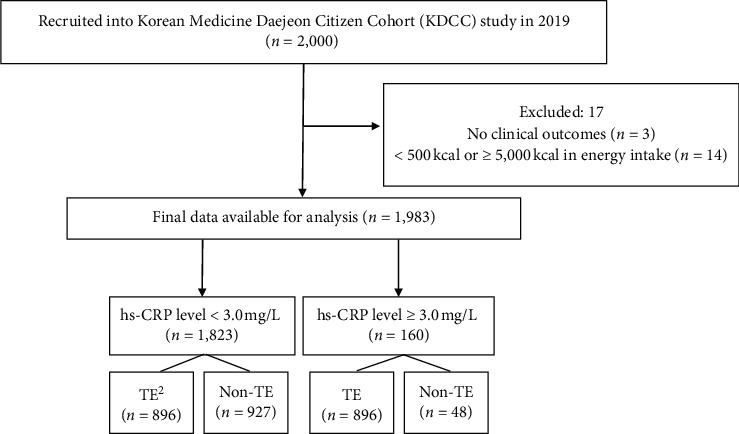
Flow diagram illustrating the selection of subjects for analysis. hs-CRP levels <3 mg/L reflect a low systemic inflammatory status and lower to moderate vascular risk; whereas, levels ≥3 mg/L indicate high systemic inflammatory status and higher vascular risk. Constitution type was categorized into two groups: TE (Tae-eumin) or non-TE (So-eumin and So-yangin).

The participants included individuals aged ≥30 years and ≤55 years who were residents of Daejeon at the time of enrollment in the KDCC between June 2017 and December 2019. Participants were excluded if they (1) had difficulty reading and understanding or following study instructions or (2) were diagnosed with cancer(s) or any CVDs, such as myocardial infarction, angina, or stroke. A total of 2,000 participants were enrolled in the study at baseline. Participants with no clinical outcomes (*n* = 3) and those with less than 500 kcal or more than 5,000 kcal energy intake (*n* = 14) were excluded from the analysis. In total, 1,983 participants were included in the present study. Tae-yang type was not included in the analysis owing to the low rates (<0.1%) of all participants. The inflammatory status of the participants was categorized into low-risk (<3.0 mg/L) or high-risk (≥3.0 mg/L) groups according to their constitution types (Figure 1).

### 2.2. Definition of Cardiometabolic Risk Factors and the hs-CRP Level

Cardiometabolic risk factors were previously categorized by the National Cholesterol Education Program-Adult Treatment Panel III (NCEP-ATP III) [26], requiring participants to meet more than two of the following clinical criteria: abdominal obesity based on waist circumference (WC) with cutoff points specific to South Koreans (WC ≥90 cm in men and ≥85 cm in women) plus any of the following: elevated triglyceride (TG) levels ≥150 mg/dL or specific treatment for this lipid abnormality; reduced high-density lipoprotein-cholesterol (HDL-C) level, < 40 mg/dL in men and < 50 mg/dL in women or drug treatment for this lipid abnormality; elevated blood pressure (BP), systolic BP (SBP) ≥130, or diastolic BP (DBP) ≥85 mmHg or treatment of previously diagnosed hypertension; and the elevated fasting plasma glucose (FPG) level ≥100 mg/dL or previously diagnosed type 2 diabetes.

The participants were categorized into two groups: (1) hs-CRP levels <3 mg/L reflecting a low systemic inflammatory status and lower to moderate vascular risk and (2) hs-CRP levels ≥3 mg/L indicating a high systemic inflammatory status and higher vascular risk [4].

### 2.3. Sasang Constitution Type

The Korean Sasang constitutional diagnostic questionnaire (KS-15) [27] was employed to assess the individual constitution of the participants. The KS-15 questionnaire consists of 15 items of anthropometric awareness of height and weight and six questions of personality and eight symptom-related questions of physiological functions (Cronbach's alpha = 0.630). The KS-15 is a well-validated short-form assessment tool for constitution type that adapts body mass index (BMI) and age- and sex-specific weighted values for higher coincidence with clinical relevance [27]. We defined the two constitution types as TE type or non-TE (So-yangin, SY and So-eumin, SE) type to compare the differences in the dietary factors related to cardiometabolic outcomes and inflammatory status among the three main types of constitution. Previous studies have showed that 20−30% of Korean adults were categorized into SY or SE types, while almost half of them had TE type. The non-TE type has opposite or different characters in their traits, diet, BMI, disease prevalence, and genetic factors when compared with that of the TE type [28–30].

### 2.4. Sociodemographic Characteristics, Anthropometric Measurements, and Biochemical Data

Sociodemographic characteristics for the study population, including age, sex, education level, household income, smoking, alcohol consumption, and physical activity, were surveyed at baseline.

Anthropometric measurements to the nearest 0.1 kg or 0.1 cm (i.e., height, weight, WC, SBP, and DBP) were taken by trained personnel with the participants dressed lightly without their shoes before height and weight measurement using the measuring station BSM 370 and InBody 770 (Biospace, Korea). BMI was calculated as weight in kg divided by height in meters squared (m^2^). BP was measured once using an automatic blood pressure cuff (FT-500R PLUS, Jawon Medica, Korea), and it was measured again after 5–10 min of rest, and the mean value of the two measurements was used for analysis. WC was measured with a tape measure (Hoechst mass-Rollfix, Germany) according to World Health Organization guidelines.

Laboratory data (i.e., TG, HDL-C, FPG, and hs-CRP) were recorded after overnight fasting. After 30 min of incubation, collected blood samples were centrifuged for 10 min at 3,450 rpm, and then, all of the samples were transported to the Seoul Clinical Laboratories (Seoul, Korea) within 24 hours. Serum TG, HDL-C, FPG, and the hs-CRP levels were measured by automated clinical analyzers (ADVIA 1800, Siemens, USA).

### 2.5. Short Form of Food Frequency Questionnaires (SF-FFQ)

Dietary intake was assessed using a semiquantitative food frequency questionnaire (FFQ), which was validated by the Korea National Health and Nutrition Examination Survey [25]. Participants were asked to report their frequency (nine options: almost null, 1 time/month, 2-3 times/month, 1-2 times/week, 3-4 times/week, 5-6 times/week, 1 time/day, 2 times/day, or 3 times/day) and portion size (three or four specified portion sizes) of each food item over the past year.

The short form of the FFQ consisted of groups of carbohydrates (rice, mixed grains, noodles, bread, potatoes, and sweet potatoes), proteins (beef, pork, fish, beans, tofu, and eggs), dairy, vegetables, fruits, seaweeds, coffee and tea, alcohol, and fast food. The reported frequency of intake for each food item was converted to daily gram intake of macronutrients as well as total energy using the computer-aided nutritional analysis program (CAN Pro, Version 5.0, The Korean Nutrition Society, 2015).

### 2.6. Statistical Analysis

Descriptive analyses of the participants according to their constitution type and MetS status are presented in Table 1. Group differences were evaluated using chi-square tests for categorical variables including age, sex, education level, household income, smoking status, alcohol consumption, and physical activity level. The results of the one-way analysis of variance and analysis of covariance (ANCOVA) for mean age, BMI, cardiometabolic risk factors, energy intake, nutrients, and food groups are given in Table 1.

We added the data to a multivariate logistic regression model to examine the association between the food frequencies and high-risk hs-CRP level (Table 2) and cardiometabolic risk factors by constitution type (Table 3). Both models (1 and 2) included several potential confounding factors such as age, sex, BMI, energy intake (kcal), smoking, alcohol consumption, and physical activity [19].

We combined vegetable consumption (lower vs. higher) and the hs-CRP level (high-risk vs. low-risk) to compare the odds ratios (ORs) of the CVD risk factors between the references (vegetable consumption >91.5 g/day and hs-CRP <3.0 mg/L) and other combinations of the two clusters (Table 4). Sociodemographic characteristics, including the education level and household income, were fully adjusted to reduce any residual confounding factors in model II.

We performed all analyses using SAS 9.4 (SAS Institute, Cary, North Carolina); all analyses were two-tailed, and a *P* value of <0.05 was considered significant.

## 3. Results

Group differences in general characteristics, CVDs risk factors, and dietary intakes of the subjects according to constitution types with hs-CRP.

A total of 1,983 participants were categorized into lower or higher than 3.0 mg/L of the hs-CRP level by their constitution types. A total of 11.1% of TE patients and 4.9% of non-TE patients had a higher hs-CRP level (TE: 6.6 ± 0.2, non-TE: 8.4 ± 0.3) than the low hs-CRP level TE and non-TE (TE: 0.9 ± 0.1, non-TE: 0.6 ± 0.1).

No significant differences were observed between the four groups in the general characteristics except for sex and smoking (*P* < 0.05).

The risk factors for CVD, BMI (kg/m^2^), WC (cm), SBP (mmHg), DBP (mmHg), FPG (mg/dL), and TG (mg/dL) were the highest (*P* < 0.0001), while HDL-C (mg/dL) was the lowest in the high TE group (*P* < 0.0001) (Table 1).

No significant group differences were found in the energy intake (kcal) or nutrient intake between the four groups. Regarding the food groups (g/d), in vegetables, mushrooms were highest in the low hs-CRP TE group and lowest in the high hs-CRP TE group (low TE: 54.3 ± 1.1 vs. high TE: 45.1 ± 3.1, *P*=0.025) after adjusting for age, sex, and energy intake (kcal).

### 3.1. Associations between Fruits, Vegetables, and Red Meats Consumption and the hs-CRP Level of the Subjects by Constitution Types

A covariate-adjusted multiple logistic regression analysis was performed to assess the beneficial or harmful association between tertiles of fruits, vegetables, and red meats and the hs-CRP level. No significant association between tertiles of fruits or meats and the hs-CRP level was observed in the TE and non-TE groups after adjusting for age, sex, BMI, energy intake (kcal) (model 1), and model 2 (model 1+ smoking, alcohol consumption, and physical activity) (Table 2).

In the tertile of vegetables consumption, an approximately 2.5-fold higher risk of hs-CRP (Hs-CRP level ≥3.0 mg/L) was seen in tertiles 1 (T1: 21.2 g/day) and 2 (T2: 50.3 g/day) groups compared with the highest group (T3: 91.5 g/day) in TE (adjusted ORs: T2: 2.290 (95% confidence interval (CI): 1.285–4.082); T1: 2.474 (95% CI: 1.368–4.475), *P*=0.003).

### 3.2. Associations of Cardiometabolic Risk Factors and Tertiles of Vegetable Consumption of the Subjects by Constitutional Types

The associations between tertiles of vegetable consumption and cardiometabolic risk factors are presented in Table 3. No statistical significance was observed in the cardiometabolic risk factors such as elevated BP, FPG, TG, reduced HDL-c, and high WC; in contrast, individuals with more than two concurrent those of CVDs risk factors showed significant associations. The multivariate-adjusted ORs of the prevalence of more than two concurrent CVDs risk factors and the lowest (T1) vegetable consumption was 1.631 (95% CI: 1.106–2.404, *P*=0.012) in TE (Table 3).

### 3.3. Multivariate Logistic Regression between Vegetable Consumption, hs-CRP Level, and Cardiometabolic Risk Factors by Constitution Types

The multivariate-adjusted regression models (models I and II) showed that higher vegetable consumption greatly decreases the prevalence of CVDs risk factors in model I (OR: 0.248, 95% CI: 0.064–0.961) and model II (OR: 0.141, 95% CI: 0.028–0.695) in the high-risk hs-CRP TE group. Similarly, significant ORs of the prevalence of CVDs risk factors were estimated in the low-risk hs-CRP TE group (Table 4).

## 4. Discussion

This study examined the association between dietary factors, hs-CRP level, and cardiometabolic risk factors in Korean adults according to their constitution types. Overall, 8.1% of the participants in the present study had high-risk hs-CRP levels. Our results illustrate that lower consumption of vegetables (<92 g/d) is highly associated with the prevalence of having a high-risk hs-CRP level (adjusted ORs: T2: 2.290 (95% CI: 1.285–4.082), T1: 2.474 (95% CI: 1.368–4.475), *P*=0.003) in the TE group. Furthermore, the lowest (T1) of vegetable consumption had a 63% increased prevalence of more than two concurrent CVD risk factors compared with that of the highest in the TE group (*P*=0.012). Last, higher vegetable consumption greatly decreased the prevalence of CVD risk factors by 63–86% in the low-risk and high-risk hs-CRP TE groups.

A previous meta-analysis reported a positive association between active smoking and MetS (RR: 1.26, 95% CI) [31]. Another cross-sectional study found an increased risk of high TGs, low HDL-C, and a decreased risk of high BP compared with nonsmokers [32]. However, this could not elucidate the interrelationship directly owing to disparities in the extent of the contribution of sex differences and inaccurate self-reported smoking status without cotinine-verified smoking status in this study. Therefore, we used smoking status with other lifestyle-related (alcohol consumption, physical activity, and energy intake) variables including age and sex as potential confounding factors for the present study.

An elevated hs-CRP level, which is the most standardized inflammatory marker, predicts the risk for developing MetS and CVDs. The association between MetS and its cluster with hs-CRP is consistent with the findings of previous studies. A Korean population-based study found that the hs-CRP level plays a major role in the development of MetS [5]. In women, obesity status affects the inflammatory status, while BMI was considered the most significant risk factor in men. Regardless of the positive association between the hs-CRP level and hyper TG, low HDL-C levels were observed. Similarly, these traditional markers including WC and lipid parameters such as TG, HDL-C, and LDL-C levels were significantly related to the hs-CRP level not only in obese females [33, 34] but also that of adolescent girls [35]. In the Bogalusa heart study [36], the elevated hs-CRP level was associated with follow-up HOMA-IR and increased risk of T2DM in nondiabetic adults. This finding suggests that there is a combined effect of inflammation and insulin resistance on the pathogenesis of hyperglycemia. In fact, a previous U.S. population study found that the elevated hs-CRP group had more CVD with MetS [37]. It has also been predicted that there is a 1.48-times higher MetS risk in the hs-CRP increased group than in that of their counterparts in healthy subjects [38].

Consistently, 39.7% of the participants had high-risk hs-CRP, and the highest prevalence of the predictors of CVD was found in the TE type [39]. A previous study found that TE type itself was a “risk factor” for an increasingly higher odds ratio (adjusted ORs: 1.99, 95% CI: 1.60–2.47) of the high-risk hs-CRP compared with non-TE types (SY or SE).

Our results support the impression of differences between the TE and non-TE groups; the TE group had a higher BMI as well as more CVDs risk factors such as WC, BP, FPG, and TG and reduced HDL-c compared with those in the non-TE group. The development of MetS in the TE group is likely due to dysregulated metabolic homeostasis underlying CVD-related pathogenesis, such as dyslipidemia or elevated BP. Hence, it is important to identify individuals who have a high risk of CVD by reducing MetS and CVD-related mortality.

Our results highlight the harmful effects of low vegetable consumption on the high-risk hs-CRP level groups as well as the cardiometabolic risk factors in the TE group. This is in agreement with a previous study that reported the association of low adherence to the traditional Mediterranean diet with inflammatory biomarkers in the adult population [40, 41]. Similarly, a large population-based study reported an inverse association between high intake of a vegetable-seafood dietary pattern and components of MetS and CRP but found a positive association between high intake of a meat-instant food dietary pattern and components of MetS and CRP [22]. Another population-based prospective study observed that a proinflammatory diet represents a potential MetS risk factor in 157,812 Korean adults (mean age 52.8 years; mean follow-up of 7.4 years) [42]. Additionally, a systematic review found that CRP and meat-based or Western-like dietary patterns were positively associated while vegetable- and fruit-based or healthy patterns were negatively associated after adjusting for potential confounders [23].

A choice of healthy foods such as fruits, vegetables, whole grains, nuts, seeds, and legumes has beneficial effects in lowering inflammation, whereas a Western dietary pattern that includes red meat with saturated fatty acids is associated with excessive production of proinflammatory cytokines in the body [20]. In the present study, no group differences were found in energy intake, macronutrients, and food groups except vegetables between the participants. This reflects, even in the metabolically high-risk type (normally, TE), that no specific problematic eating related to inflammation was observed in their diet. Therefore, future studies are needed to assess the dietary risk factors for secondary prevention in the development of MetS or CVD.

Importantly, obesity-induced metabolic inflammation produces CRP, as a proinflammatory biomarker, and adipokines, including tumor necrosis factor-alpha (TNF-*α*) and interleukin (IL)-6, which further propagate inflammation [43]. An inappropriate diet, such as high sugar-sweetened beverages and low fruit and vegetable intake, is related to the development of a proinflammatory state, which leads to cardiometabolic diseases [44, 45]. In contrast, an anti-inflammatory nutrient-based diet has complex dietary bioactive properties, such as fiber, polyphenols, and vitamins that promote anti-inflammatory and cardioprotective effects [46, 47]. In fact, a higher fiber intake (≥15 g/1,000 kcal) was associated with lower HbA1c and CRP than lower fiber intake in 1,785 middle-aged people with type 2 diabetes [48]. Furthermore, a significant inverse association was observed between total vegetable consumption and TNF-*α* level (−0.078; 95% CI, −0.151 to −0.005) in obese older adults [24]. Another previous study found that 7.1% weight loss, 22.3% visceral adiposity reduction, 15.5% improvement in HOMA-IR and lipid profiles, and inflammatory biomarkers (hs-CRP, −29.5%; IL-6, −18.2%; TNF-*α*, −34.2%) following energy-restricted anti-inflammatory diet therapy for patients with nonalcoholic fatty liver disease [49].

In line with this, we found a significant protective effect of high vegetable consumption on cardiometabolic risk factors in the high-risk and low-risk hs-CRP level TE groups. Several large population-based studies have reported that there is an inherent metabolic high-risk associated with the components of MetS in TE [9–14]. In particular, high BMI and obesity-related metabolic risks, such as central obesity (WC and WHR) [9, 11, 50], which are interrelated to the development of insulin resistance [14] and T2DM [15], were found in the TE group. Obesity is known to produce a chronic low-grade inflammatory status, which is the pathogenesis of MetS and the progression of CVD. For this reason, early detection and specific treatment of CVDs are essential for patients with obesity.

The main strengths of our study are that it uses data from a nationally representative survey of Korean adults and that it is the first study to investigate associations between dietary risk factors related to the inflammatory status and CVD risk factors by constitution types. Nevertheless, we detected only <10% of the high-risk level of hs-CRP in the present study samples (between unequal sample size); therefore, one needs to be careful when interpreting the data. This study also had several limitations. First, the design of this study was cross-sectional, permitting to make projections to the odds of the prevalence of CVD. Second, we used only serum hs-CRP levels to assess the inflammatory status. Third, we employed a short form of the FFQ to assess dietary factors for the participants' convenience. Except for vegetable consumption, we could not find any other beneficial or harmful dietary factors related to the hs-CRP level or CVD risk factors in Korean adults. Further research is warranted to evaluate overall diet or dietary patterns to explore diet-related cardiometabolic risk factors in the inherent metabolically high-risk Korean population.

## 5. Conclusions

In summary, this study demonstrates the existence of a significant relationship between the dietary factors, hs-CRP level, and cardiometabolic risk factors in Korean adults based on the constitution types. The present data suggest that higher consumption of vegetables provides cardioprotective effects in Korean adults with TE. Evidence-based clinical risk factor management and multifaceted approaches at the community and population levels targeting prevention in high-burden groups are strongly needed to reduce premature mortality from CVD.

## Figures and Tables

**Table 1:** Group differences in general characteristics, CVDs risk factors and dietary intakes of the subjects according to constitution type† with high-sensitive CRP level‡.

hs-CRP level, mg/L		hs-CRP level <3.0 mg/L (*n* = 1823)		hs-CRP level ≥3.0 mg/L (*n* = 160)		*P* value
Characteristics		TE (*n* = 896)	Non-TE (*n* = 927)	TE (*n* = 112)	Non-TE (*n* = 48)	
Age (years), (mean ± SE)		44.2 ± 0.2^a^	44.8 ± 0.2^a^	**42.3 ± 0.6** ^**b**^	**43.7 ± 1.0** ^**ab**^	**0.001**
30–44 years (*n*, %)		447 (49.9)	448 (48.3)	**71 (63.4)**	28 (58.3)	**0.016**
45–55 years		274 (50.1)	385 (51.7)	41 (36.6)	20 (41.7)	

Sex (%)						
Male		339 (37.8)	222 (23.9)	31 (27.7)	13 (27.1)	**<0.0001**
Female		557 (62.2)	**705 (76.1)**	81 (72.3)	35 (72.9)	

Education (%)						0.977
High school lower levels		318 (35.7)	330 (35.7)	42 (37.8)	17 (36.2)	
College and higher levels		572 (64.3)	594 (64.3)	69 (62.2)	30 (63.8)	

Household income^1^ (%)						0.977
Low		170 (19.1)	157 (17.2)	24 (21.4)	7 (14.6)	
Middle		652 (73.2)	662 (72.4)	77 (68.8)	35 (72.9)	
High		69 (7.7)	96 (10.5)	11 (9.8)	6 (12.5)	

Smoking (%)						0.030
No		777 (86.7)	824 (88.9)	93 (83.0)	47 (97.9)	** **
Yes		119 (13.3)	103 (11.1)	19 (17.0)	1 (2.1)	

Alcohol consumption (%)						0.125
No		195 (35.3)	320 (41.4)	93 (83.0)	47 (97.9)	
Yes		**358 (64.7)**	452 (58.6)	19 (17.0)	1 (2.1)	

Physical activity^2^ (%)						
Low		275 (30.7)	274 (29.6)	23 (20.5)	18 (37.5)	0.067
Moderate		270 (30.1)	297 (32.0)	45 (40.2)	19 (39.6)	
High		351 (39.2)	356 (38.4)	44 (39.3)	11 (22.9)	
BMI, kg/m^2§^		26.7 ± 0.1^b^	22.2 ± 0.1^c^	**29.3** ± **0.2**^**a**^	22.5 ± 0.4^c^	**<0.0001**

CVDs risk factors (mean ± SE)^§^						
WC, cm		88.6 ± 0.2^b^	78.6 ± 0.2^c^	**94.5 ± 0.5** ^**a**^	79.8 ± 1.0^c^	**<0.0001**
Systolic BP, mmHg		121.3 ± 0.5^b^	115.6 ± 0.5^c^	**124.7 ± 1.4** ^**a**^	115.7 ± 2.0^c^	**<0.0001**
Diastolic BP, mmHg		77.4 ± 0.5^a^	73.0 ± 0.4^b^	**78.7 ± 1.0** ^**a**^	72.4 ± 1.6^b^	**<0.0001**
Fasting plasma glucose, mmol/L		**86.6 ± 0.5** ^**a**^	83.0 ± 0.6^b^	**89.6 ± 1.5** ^**a**^	85.6 ± 2.3^ab^	**<0.0001**
HDL-C, mmol/L		52.2 ± 0.4^b^	58.7 ± 0.4^a^	**48.2 ± 1.2** ^**c**^	52.8 ± 1.8^bc^	**<0.0001**
TG, mmol/L		156.7 ± 3.2^ab^	130.2 ± 3.4^c^	**177.3 ± 9.1** ^**a**^	129.9 ± 13.8^bc^	**<0.0001**
hs-CRP level, mg/L		0.9 ± 0.1^c^	0.6 ± 0.1^d^	**6.6 ± 0.2** ^**b**^	**8.4 ± 0.3** ^**a**^	**<0.0001**
≥2 concurrent CVDs risk factors		442 (49.3)	143 (15.4)	**77 (68.8)**	10 (20.8)	**<0.0001**

Energy (kcal/d)^3^						
Men		2238.5 ± 35.9	2283.6 ± 44.5	2244.5 ± 118.8	2409.4 ± 183.7	0.724
Women		2082.2 ± 29.4	2038.8 ± 26.2	2117.0 ± 77.4	2253.8 ± 117.4	0.211

Nutrients						
Carbohydrates (g)		314.9 ± 7.3	321.0 ± 4.9	313.1 ± 5.3	339.9 ± 8.1	0.828
Fat (g)		53.9 ± 1.7	53.9 ± 1.1	51.6 ± 1.0	53.8 ± 1.7	0.418
Protein (g)		72.3 ± 1.9	72.5 ± 1.2	69.6 ± 1.2	73.6 ± 1.9	0.636
C : F : P (%)		59.8 : 22.7 : 13.6	59.8 : 22.5 : 13.5	60.6 : 22.5 : 13.4	60.9 : 21.9 : 13.4	N/S
Food groups (g/d)^4§§^						
White rice		191.7 ± 2.6	193.4 ± 2.7	183.7 ± 7.3	205.4 ± 11.1	0.385
Wholegrains		78.5 ± 2.7	74.9 ± 2.8	88.7 ± 7.6	84.5 ± 11.0	0.290
Noodles and bread		102.4 ± 3.1	108.3 ± 3.2	96.8 ± 8.8	91.3 ± 13.3	0.284
Potatoes and sweet potatoes		46.4 ± 2.4	45.9 ± 2.5	37.5 ± 6.9	48.4 ± 10.5	0.662
Beans and tofu		28.5 ± 1.0	26.9 ± 1.0	28.0 ± 2.8	25.0 ± 4.2	0.622
Fish		6.5 ± 0.3	6.3 ± 0.3	5.0 ± 0.8	6.5 ± 1.1	0.285
Beef pork		60.1 ± 1.9	57.4 ± 2.0	56.1 ± 5.3	58.8 ± 8.1	0.739
Poultry		34.7 ± 1.4	31.9 ± 1.5	28.7 ± 4.0	31.1 ± 6.1	0.347
Eggs		36.7 ± 1.2	34.9 ± 1.3	33.8 ± 3.4	29.7 ± 5.2	0.445
Vegetables and mushrooms		**54.3 ± 1.1** ^**a**^	52.5 ± 1.1^ab^	**45.1 ± 3.1** ^**b**^	57.6 ± 4.7^ab^	**0.025**
Fruits		84.7 ± 3.4	91.0 ± 3.5	73.5 ± 9.5	77.3 ± 14.2	0.214
Milk and yogurt		101.4 ± 3.8	102.3 ± 4.0	119.3 ± 10.9	100.3 ± 16.4	0.481
Coffee and Tea		125.2 ± 3.6	123.4 ± 3.7	136.5 ± 9.9	122.4 ± 15.4	0.659
Beer and soju		45.3 ± 2.6	53.4 ± 2.7	47.6 ± 7.3	39.2 ± 10.7	0.111
Hamburger and pizza		88.4 ± 4.5	88.4 ± 4.5	95.8 ± 12.6	94.1 ± 19.7	0.771

^†^Constitution type was categorized into two groups as TE (Tae-eumin) and non-TE (So-eumin and So-yangin). ^‡^High-sensitive CRP levels were categorized into two different groups: hs-CRP level < 3.0 mg/L and hs-CRP level ≥ 3.0 mg/L. ^1^Monthly household income was divided into 3 groups: low (> 3,000,000 won), middle (3,000,000–7,000,000 won), and high (7,000,000 won ≤). ^2^Physical activity: total MET minutes per week: low (<600 MET-min/wk); moderate (600–1,500 MET-min/wk); and high (1,500 MET-min/wk). *P* values were obtained from Rao-Scott chi-square tests for categorical variables and Bonferroni multiple comparison of one-way analysis of variance and analysis of covariance (ANOVA). ^a^The different letters indicate statistically significant differences (*P* < 0.05), analyzed using ANCOVA followed by Bonferroni's multiple comparision test. ^§^Least square means-sq adjusted for age and sex. CVDs, cardiovascular diseases; hs-CRP, high-sensitive C-reactive protein; BMI, body mass index; WC, waist circumference; BP, blood pulse; HDL-C, high-density lipoprotein cholesterol; TG, triglycerides. ^3^Adjusted age only. ^§§^Least square means-sq adjusted for age, sex, and energy intake (kcal). ^4^Food groups were surveyed using short-form of the food frequency questionnaires (FFQ) which includes grains (white rice, wholegrains, noodles bread, potatoes, and sweet potatoes) proteins (bean, tofu, fish, beef, pork, poultry, and eggs); vegetables (cabbage, radish, carrot, zucchini, and mushrooms); fruits (apples, banana, persimon, strawberries, and pear); dairy (milk and yogurt); drink (coffee and tea); alcohol (beer and soju); fast foods (hamburger and pizza).

**Table 2:** Associations between fruits, vegetables, fish and meats consumptions and high-risk hs-CRP level of the subjects by constitution types.

Food groups	hs-CRP level ≥3.0 mg/L	Model 1 OR (95% CI)		Model 2 OR (95% CI)	
		TE	Non-TE	TE	Non-TE
Fruits					
Adjusted OR (95% CI)		(Ref = T3: 155.5 g/day)		(Ref = T3: 155.5 g/day)	
	T2: 59.6 g/day	1.116 (0.658 – 1.893)	0.911 (0.458 – 1.811)	1.158 (0.673 – 1.994)	0.976 (0.472 – 2.018)
	T1: 17.8 g/day	0.940 (0.543 – 1.627)	0.712 (0.327 – 1.549)	0.976 (0.552 – 1.727)	0.899 (0.400 – 2.019)
*P* value for trend		0.923	0.441	0.954	0.822

Vegetables					
Adjusted OR (95% CI)		(Ref = T3: 91.5 g/day)		(Ref = T3: 91.5 g/day)	
	T2: 50.3 g/day	**2.169 (1.235-3.809)**	0.651 (0.320 – 1.322)	**2.290 (1.285** – **4.082)**	0.645 (0.308 – 1.350)
	T1: 21.2 g/day	**2.488 (1.401-4.421)**	0.634 (0.300 – 1.339)	**2.474 (1.368** – **4.475)**	0.564 (0.257 – 1.236)
*P* value for trend		**0.002**	0.210	**0.003**	0.140

Fish					
Adjusted OR (95% CI)		(Ref = T3: 11.4 g/day)		(Ref = T3: 11.4 g/day)	
	T2: 4.0 g/day	1.578 (0.914 – 2.724)	1.069 (0.518 – 2.205)	1.664 (0.948 – 2.921)	1.043 (0.489 – 2.226)
	T1: 1.9 g/day	1.564 (0.900 – 2.718)	0.931 (0.445 – 1.947)	1.562 (0.882 – 2.769)	0.948 (0.436 – 2.063)

*P* value for trend		0.080	0.931	0.080	0.948
Beef and pork					

Adjusted OR (95% CI)		(Ref = T3: 87.0 g/day)		(Ref = T3: 87.0 g/day)	
	T2: 39.1 g/day	1.190 (0.695 – 2.039)	0.777 (0.367 – 1.644)	1.090 (0.627 – 1.894)	0.798 (0.363 – 1.752)
	T1: 17.4 g/day	0.922 (0.523 – 1.626)	0.645 (0.290 – 1.435)	0.836 (0.465 – 1.504)	0.634 (0.269 – 1.493)
*P* value for trend		0.934	0.277	0.668	0.305

Model I, age, sex, BMI, and energy intake (kcal). Model II: model I + smoking, alcohol consumption, and physical activity. Boldface type indicates statistical significance.

**Table 3:** Associations of cardiometabolic risk factors and tertiles of the vegetables consumption of the subjects by constitution types.

CVDs risk factors		Vegetables consumption			
		Model 1 OR (95% CI)		Model 2 OR (95% CI)	
		TE	Non-TE	TE	Non-TE
High BP (SBP ≥ 130 or DBP ≥85 mmHg)		(Ref = T3: 91.5 g/day)			
	T2	1.397 (0.984 – 1.983)	0.868 (0.554 – 1.360)	1.370 (0.956 – 1.963)	0.862 (0.544 – 1.365)
	T1	1.254 (0.874 – 1.801)	1.412 (0.909 – 2.193)	1.258 (0.868 – 1.823)	1.389 (0.880 – 2.180)
*P* value for trend		0.211	0.119	0.219	0.150
Elevated FPG (≥100 mg/dL)		(Ref = T3: 91.5 g/day)			
	T2	1.465 (0.881 – 2.436)	0.804 (0.326 – 1.988)	1.414 (0.837 – 2.389)	0.803 (0.322 – 2.002)
	T1	0.853 (0.483 – 1.506)	1.416 (0.608 – 3.301)	0.789 (0.435 – 1.431)	1.405 (0.595 – 3.319)
*P* value for trend		0.655	0.424	0.506	0.436
Elevated TG (≥150 mg/dL)		(Ref = T3: 91.5 g/day)			
	T2	1.146 (0.816 – 1.610)	0.809 (0.512 – 1.279)	1.165 (0.819 – 1.657)	0.856 (0.535 – 1.369)
	T1	1.051 (0.740 – 1.493)	1.273 (0.805 – 2.014)	1.130 (0.786 – 1.623)	1.317 (0.818 – 2.119)
*P* value for trend		0.768	0.294	0.502	0.252
Reduced HDL-C (< 40 mg/dL in men and < 50 mg/dL in women)		(Ref = T3: 91.5 g/day)			
	T2	1.017 (0.715 – 1.448)	0.929 (0.604 – 1.431)	1.146 (0.795 – 1.652)	1.0732 (0.684 – 1.683)
	T1	1.118 (0.779 – 1.602)	0.745 (0.466 – 1.191)	1.238 (0.852 – 1.799)	0.841 (0.515 – 1.372)
*P* value for trend		0.549	0.223	0.261	0.503
High WC (≥90 cm in men and ≥85 cm in women)		(ref = T3: 91.5 g/day)			
	T2	1.186 (0.797 – 1.765)	1.059 (0.481 – 2.333)	1.254 (0.832 – 1.890)	0.864 (0.379 – 1.967)
	T1	1.166 (0.774 – 1.758)	1.309 (0.600 – 2.855)	1.259 (0.824 – 1.924)	0.989 (0.434 – 2.254)
*P* for trend		0.453	0.490	0.278	0.997
≥2 concurrent CVDs risk factors		(Ref = T3: 91.5 g/day)			
	T2	**1.468 (1.023 – 2.109)**	0.906 (0.564 – 1.455)	**1.542 (1.061 – 2.243)**	0.953 (0.587 – 1.550)
	T1	**1.541 (1.058 – 2.244)**	1.099 (0.677 – 1.787)	**1.631 (1.106 – 2.404)**	1.114 (0.673 – 1.844)
*P* value for trend		**0.023**	0.709	**0.012**	0.679

Model I, age, sex, BMI, and energy intake (kcal). Model II, model I + smoking, alcohol consumption, and physical activity. Boldface type indicates statistical significance.

**Table 4:** Multivariate logistic regression between vegetables consumption, hs-CRP level and cardiometabolic risk factors by constitution type.

	Vegetables consumption	hs-CRP level	≥ 2 concurrent CVDs risk factors	
			TE	Non-TE
Model I OR (95% CI)	(Ref, <91.5 g/day)	(Ref, ≥3.0 mg/L)	—	
	Lower	Low-risk	0.553 (0.305–1.006)	0.501 (0.178–1.406)
	Higher	High-risk	**0.248 (0.064**–**0.961)**	0.724 (0.123–4.275)
	Higher	Low-risk	**0.368 (0.197**–**0.691)**	0.434 (0.141–1.339)

Model II OR (95% CI)	(Ref, <91.5 g/day)	(Ref, ≥3.0 mg/L)	—	
	Lower	Low-risk	0.557 (0.305–1.018)	0.625 (0.206–1.895)
	Higher	High-risk	**0.141 (0.028**–**0.695)**	0.695 (0.054–8.948)
	Higher	Low-risk	**0.361 (0.190**–**0.684)**	0.548 (0.161–1.859)

Model I, age, sex, BMI, energy intake (kcal), smoking, alcohol consumption, and physical activity. Model II, model I + education level and household income. Boldface type indicates statistical significance.

## Data Availability

The data that support the findings of this study are available from the web-based KDC electronic data capture system by the Korean Medicine Data Center (KDC) of the KIOM, but restrictions apply to the availability of these data, which were used under license for the current study, and so are not publicly available.
